# An Optimal Screw Fixation Strategy for Posterior Malleolar Fractures: A Systematic Review and Meta-Analysis Comparing Posteroanterior Versus Anteroposterior Screws

**DOI:** 10.7759/cureus.91221

**Published:** 2025-08-29

**Authors:** GuiSong Yu, FengBing Hu, YuBo Cui, YuPeng Dong, Wei Liang, GuangYi Zou, Xing Fu, Dian Li, WenLong Yang, FengYun Yang

**Affiliations:** 1 Orthopedics, Jiangxi University of Traditional Chinese Medicine School of Clinical Medicine, Nanchang, CHN; 2 Orthopedics and Trauma, Lishui People’s Hospital, Lishui, CHN; 3 Orthopedics, Changxing County Hospital of Traditional Chinese Medicine, Huzhou, CHN; 4 Traumatology and Orthopedics, Lishui People’s Hospital, Lishui, CHN; 5 Orthopedics, Affiliated Hospital of Jiangxi University of traditional Chinese Medicine, Nanchang, CHN; 6 Orthopedics, Affiliated Hospital of Jiangxi University of Traditional Chinese Medicine, Nanchang, CHN

**Keywords:** anterior-to-posterior screws, meta-analysis, posterior ankle fracture, posterior-to-anterior screws, review

## Abstract

The most appropriate fixation trajectory for posterior malleolar fractures (PMFs) still remains a matter of debate. This meta-analysis aimed to evaluate and compare the outcomes of posteroanterior (PA) and anteroposterior (AP) screw fixation strategies. We conducted a systematic review and meta-analysis following Preferred Reporting Items for Systematic Reviews and Meta-Analyses (PRISMA) guidelines. Both manual and electronic searches of multiple databases were conducted. Two independent reviewers screened the studies in accordance with the predefined PICOS criteria: Population (adult patients diagnosed with PMFs); Intervention (PA screw fixation); Comparison (AP screw fixation); Outcome (radiological parameters, screw malpositioning error, American Orthopaedic Foot and Ankle Society (AOFAS) scores, postoperative complications); and Study Design (evidence levels I-III). Statistical synthesis was carried out using Stata 15.1 software (StataCorp LLC, College Station, Texas, USA). A total of 14 articles were included, encompassing 1258 study participants. The findings revealed that there were no statistically significant differences in the postoperative complication rates between the posterior-anterior screw fixation technique and the anterior-posterior screw fixation technique for the management of posterior ankle fractures. Nevertheless, statistically significant differences were observed in the AOFAS scores, the incidence of screw malpositioning, and the radiographic parameters with displacement exceeding 2 mm (P < 0.05). However, the clinical relevance of the difference in AOFAS scores is uncertain. In comparison to the AP method, PA screw fixation yielded superior radiographic outcomes, higher implant accuracy, and better functional recovery as evaluated by the AOFAS scores. The choice of technique should be individualized based on fracture pattern and surgeon expertise.

## Introduction and background

The ankle joint is a crucial weight-bearing joint, and the integrity of the posterior malleolus is vital for its stability and function. The incidence of posterior malleolar fractures (PMFs) among all ankle fractures is high. The ankle joint is a crucial weight-bearing joint of the lower limb. Its stability exerts a pivotal influence on the locomotor and weight-bearing functions of the lower limb. Structurally, it mainly comprises the articular surface of the distal tibia, the articular surface of the inferior end of the fibula, and the talar trochlea [1]. The posterior malleolus, as an integral component of the ankle joint, plays a vital role in the load transfer of the tibio-talar joint, the stability of the posterior talus, and the rotational stability of the ankle joint, given its integrity and the attachment sites of its ligaments [2]. The incidence rate of PMFs among all ankle fractures is as high as 7% to 44% [3]. Evidence from previous studies has indicated that ankle fractures involving the posterior malleolus are more prone to causing joint instability and are associated with a poorer prognosis [4-6]. Consequently, surgical intervention represents the optimal approach for improving the prognosis [7]. In the treatment of PMFs, methods such as plate fixation, posterior-to-anterior screw fixation, and anterior-to-posterior screw fixation have been proposed, yet a consensus has not been reached thus far [8-16].

In recent years, there has been an ongoing debate regarding whether "anterior-to-posterior" or "posterior-to-anterior" screw placement is more appropriate for the treatment of PMFs. Although direct posteroanterior (PA) screw fixation may facilitate reduction, concerns about soft tissue injury persist. Conversely, indirect anteroposterior (AP) screw fixation minimizes soft tissue dissection but may risk suboptimal reduction. The current body of evidence is characterized by a predominance of retrospective studies and a lack of high-quality randomized controlled trials (RCTs). Therefore, this article systematically collects the relevant literature and conducts a meta-analysis on the clinical efficacy and safety of these two surgical procedures for the treatment of PMFs.

## Review

Materials and methods

Search Strategy

This meta-analysis adhered to the PRISMA (Preferred Reporting Items for Systematic Reviews and Meta-Analyses) guidelines [17]. The data sources were retrieved from PubMed, Web of Science, Embase, Wanfang Database, and China National Knowledge Infrastructure (CNKI). The search terms used were: ("ankle fracture") AND ("bone screw"). This search strategy was designed to be specific but may not have captured all relevant synonyms.

Inclusion and Exclusion Criteria

Inclusion criteria were as follows: (i) Study Subjects: Patients diagnosed with posterior malleolar fractures based on imaging findings and confirmed clinically; (ii) Interventions: The interventions involved anterior-to-posterior screw fixation and posterior-to-anterior screw fixation for posterior malleolar fractures; (iii) Outcomes: Primary outcome: Imaging assessment (Radiological evaluation (with a measurement > 2 mm)), incidence of screw malpositioning. Secondary outcome: postoperative complications (calculated as the complication rate), American Orthopaedic Foot and Ankle Society (AOFAS) Ankle-Hindfoot Score; (iv) Study Design: Randomized controlled trials or case-control studies; (v) Language: Articles written in either Chinese or English. This language restriction is acknowledged as a potential limitation.

Exclusion criteria: Duplicate publications; literature studies lacking relevant original data or for which the data are inaccessible; case reports, conference abstracts, lectures, and non-clinical studies; studies with a Newcastle-Ottawa Scale (NOS) score below 7 were excluded.

A score of 7 or higher on the NOS was predetermined to indicate satisfactory methodological quality for inclusion, a common threshold used in systematic reviews.

Data Extraction and Quality Assessment

After duplicate elimination, three independent reviewers (G.S.Y., F.B.H., and Y.B.C.) appraised the pertinence of titles and abstracts to the study's aims for each article from peer-reviewed journals. From the establishment of the respective databases up to March 2025, no journals were excluded. The results of the study screening process were presented in the subsequent table. Articles without abstracts or with non-probative information were excluded from the screening. Independent reviewers carried out a meticulous full - text perusal of the selected articles to retrieve data and reduce selection bias. Owing to the authors' restricted language capabilities, only English and Chinese articles were examined. Disputes among researchers regarding study inclusion were adjudicated by the senior researcher (F.Y.Y.), who made the conclusive decision. Ultimately, to avert potential bias, all authors reviewed, appraised, and deliberated on the selected articles, reference lists, and excluded articles.

To guarantee evaluation uniformity, six evaluators (Y.P.D., W.L., G.Y.Z., F.Y., D.L., and W.L.Y.) pre - established the data extraction protocol. For each article included in the study, the following data were retrieved: authors, publication year, geographical region, study design, sample size, fracture classification, gender, mean age, follow - up duration, and clinical outcomes (imaging assessment (radiological evaluation with a measurement > 2 mm), screw malpositioning incidence, postoperative complications, American Orthopaedic Foot and Ankle Society (AOFAS) Ankle - Hindfoot Score). We evaluated the risk of bias, encompassing selection bias (concealment of randomized sequence generation and allocation), performance bias (blinding of participants and researchers), detection bias (blinding of outcome assessors), attrition bias (incomplete outcome data), reporting bias (selective reporting), and other potential bias sources [18].

We gauged the quality of the included literature using the NOS [19]. The NOS comprises three sets of evaluations centered on: study population selection, between - group comparability, and outcome assessment. A score of 7 or higher was deemed indicative of high-quality literature, with a maximum score of 9 [20]. The inter-rater agreement for quality assessment was high.

Statistical Analysis

We carried out meta-analysis utilizing Stata 15.1 software and conducted cross-validation with RevMan 5.4 software. In dealing with continuous variables, we employed the weighted mean difference (WMD) along with its 95% confidence interval (CI). For dichotomous variables, the odds ratio (OR) together with the 95% confidence interval (95% CI) was utilized.

Owing to the disparities in research methodologies and characteristics among various studies, certain heterogeneity was observed in the outcome metrics. To evaluate the heterogeneity between the outcomes of different studies, we applied the I² statistic [21]. When the heterogeneity was deemed low, characterized by an I² value less than or equal to 50% and a p-value greater than 0.1, a fixed-effects model was adopted for the meta-analysis. Conversely, when the heterogeneity was high, with an I² value exceeding 50%, a random-effects model was employed.

For outcome indicators that exhibited any degree of heterogeneity (I² > 0%), sensitivity analyses were conducted using Stata software. In cases where the number of included papers was greater than or equal to 10, funnel plots were constructed, and Begg's and Egger's tests were carried out to assess the presence of publication bias. For studies reporting medians and interquartile ranges, data were transformed to means and standard deviations for pooling, which may introduce some imprecision. However, according to the results of subsequent sensitivity analysis, removing each study individually did not change the outcome, suggesting that the results remain stable.

Results

Literature Screening

**Figure 1:**
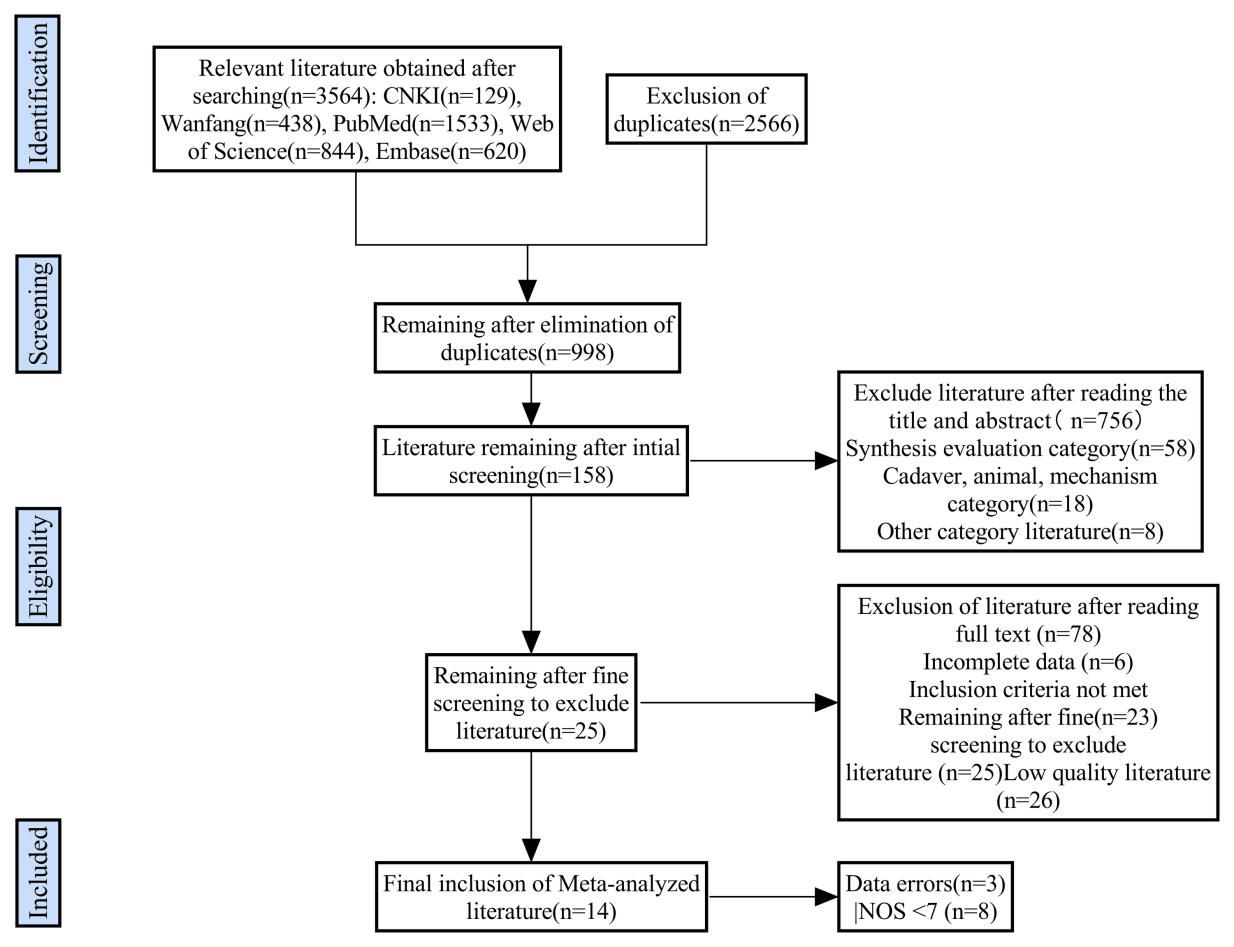
Flow diagram of the study selection process

A total of 3,564 documents were retrieved following the predefined search strategy. Among these, 2,566 duplicates were removed using Endnote software and manual duplicate checking. Further screening of titles and abstracts excluded 840 articles unrelated to the research topic. After meticulous full-text review, 14 studies were ultimately included [22-35]. The study enrolled 1,258 patients with posterior ankle fractures, including 670 patients in the PA screw fixation group and 588 patients in the AP screw fixation group. The literature screening process is depicted in Figure 1.

General Information and Quality Assessment of the Included Literature

This analysis incorporated 14 studies, encompassing three RCTs and 11 retrospective case-control studies, which compared the clinical outcomes between PA screw fixation and AP screw fixation for PMFs. A total of 1,258 patients were involved in these studies. Specifically, 670 cases underwent PA screw fixation, while 588 cases received AP screw fixation.

For each of these studies, we meticulously extracted data including the authors, publication year, geographical region, study design, sample size, fracture classification (using relevant orthopedic classification systems, such as the Lauge-Hansen classification, Haraguchi classification, AO (Arbeitsgemeinschaft für Osteosynthesefragen) classification, and other classification systems), gender, mean age, follow-up duration, and detailed clinical outcomes. The clinical outcomes covered aspects such as imaging assessments (e.g., radiological evaluations for fracture alignment and healing), incidence of screw malpositioning, postoperative complications (documented with specific medical terminologies related to orthopedic post-operative issues), and American Orthopaedic Foot and Ankle Society (AOFAS) Ankle-Hindfoot Scores.

Furthermore, we evaluated the quality of the included literature by applying the NOS score. The baseline characteristics of these studies, along with their respective NOS scores, are presented in Table 1 and Table 2 for comprehensive reference and assessment.

**Table 1:** Characteristics of the included studies *RCT: Randomized clinical trial; RCS: retrospective cohort study; PA (posteroanterior screw): posterior-anterior screw; AP (anteroposterior screw): anterior-posterior screw

Study Citation	Study design	Area	Subgroup	PA (n)	AP (n)	Mean age (PA, year)	Mean age (AP, year)	Mean age (Combined, year)	Male (n)	Female (n)
Xu et al. 2012 [22]	RCS	China	-	23	19	-	-	-	-	-
Haojun et al. 2020 [23]	RCS	China	-	32	26	44.07±12.84	41.69±13.53	-	38	20
Shengli et al. 2021 [24]	RCS	China	-	45	38	47.13±15.76	48.61±16.38	-	33	50
Chengwei et al. 2021 [25]	RCS	China	-	38	42	56.3±8.1	55.5±10.7	-	36	44
Shaoyuan et al. 2018 [26]	RCS	China	-	41	41	48.62±7.65	49.05±7.26	-	45	37
Xiunian et al. 2021 [27]	RCS	China	-	26	22	51.57±8.20	52.25±7.60	-	20	28
Jing et al. 2017 [28]	RCS	China	-	29	25	39.17±12.02	41.96±14.15	-	24	30
Vidovic et al. 2017 [29]	RCT	Croatia	-	24	22	52.3	52.3	-	28	18
Wang 2020 et al. [30]	RCS	China	＞15%	48	42	42.1±11.9	46.6±12.3	44.2±14.1	66	70
Wang 2020 et al. [30]	RCS	China	＜15%	41	31	43.2±11.9	43.8±11.8	45.8±13.4	54	53
Kalem 2018 et al. [31]	RCS	Turkey	-	13	20	48.3	43.4	-	27	40
Yu 2021 et al. [32]	RCT	Hong Kong, China	-	36	40	47.7±13.8	46.8±13.3	-	49	27
Yuanhui 2018 et al. [33]	RCT	China	-	35	34	38.1±15.8	36.8±16.7	-	46	23
Tianyi 2024 et al. [34]	RCS	China	＞17mm	121	88	42.1±12.8	-	-	110	99
Tianyi 2024 et al. [34]	RCS	China	＜17mm	93	74	-	41.9±13.0	-	87	80
Hua 2020 et al. [35]	RCS	China	-	25	24	45.51±12.16	45.62±11.97	-	28	21

**Table 2:** Characteristics of the included studies and NOS scores *Outcomes: I: Imaging assessment (Radiological evaluation (with a measurement > 2 mm)) II: incidence of screw malpositioning III: postoperative complications (calculated as the complication rate) IV: American Orthopaedic Foot and Ankle Society (AOFAS) Ankle-Hindfoot Score

Study Citation	Follow-up (month) - PA	Follow-up (month) - AP	Fracture type	Outcomes	NOS Score
Xu et al. 2012 [22]	6.9 ~ 102.3	6.9 ~ 102.3	Trimalleolar fracture	IV	7
Haojun et al. 2020 [23]	19.2±5.1	18.9±4.7	Haraguchi-I-III	II, III, IV	7
Shengli et al. 2021 [24]	15.17±5.21	16.13±5.31	Haraguchi-I	I, III, IV	7
Chengwei et al. 2021 [25]	12	12	Haraguchi-I	III, IV	7
Shaoyuan et al. 2018 [26]	36	36	Trimalleolar fracture	I, III, IV	7
Xiunian et al. 2021 [27]	13±3.50(10~20)	13±3.50(10~20)	Lauge-Hansen: SER/PER	III, IV	7
Jing et al. 2017 [28]	27.93±8.19	28.24±8.91	AO/OTA:44B/44C	II, III, IV	7
Vidovic et al. 2017 [29]	20.5(12-29)	20.5(12-29)	AO: 44B/44C	I, III, V	9
Wang et al. 2020 [30]	18.2(14.5,23.6)（＞15%）	17.7(14.4,22.3)（＞15%）	Lauge-Hansen: SER/PER	IV, V	8
Wang et al. 2020 [30]	17.5(13.9,24.2)（＜15%）	17.7(15.1,20.9)（＜15%）	Lauge-Hansen: SER/PER	IV, V	8
Kalem et al. 2018 [31]	16.3±2.56	14.4±2.23	Lauge-Hansen: SER/SA/PER/PA	III, IV	8
Yu et al. 2021 [32]	30(24-42)	30(24-42)	Haraguchi-I	I, V	9
Yuanhui et al. 2018 [33]	12.6(8.5-24)	12.6(8.5-24)	AO: 44B/44C	I, III, V	8
Tianyi et al. 2024 [34]	19.3(12-85)	19.3(12-85)	AO: 44A3/44B3/44C	IV	7
Hua et al. 2020 [35]	17.61±3.85	17.61±3.85	Haraguchi-I	I, III, IV, V	7

Assessment of Study Bias Risk

**Figure 2:**
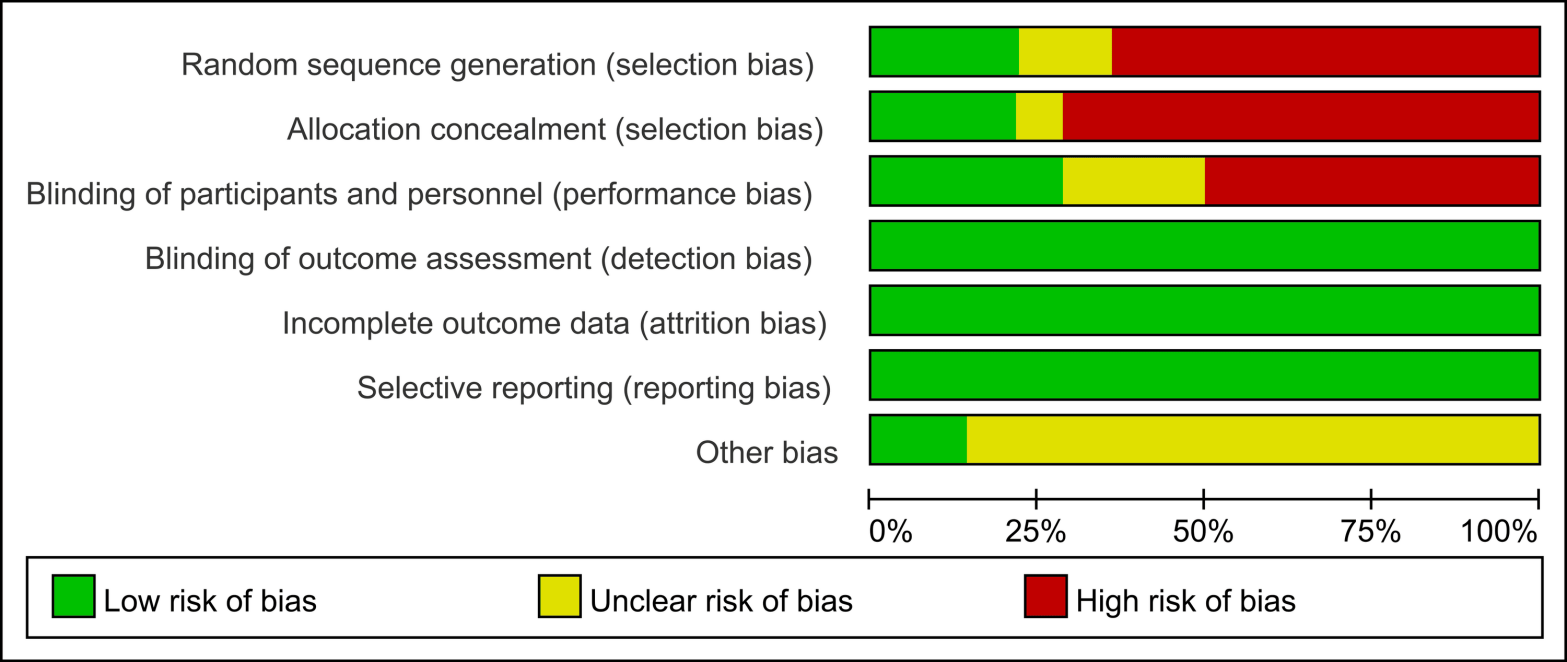
Risk of bias evaluation of included studies

**Figure 3:**
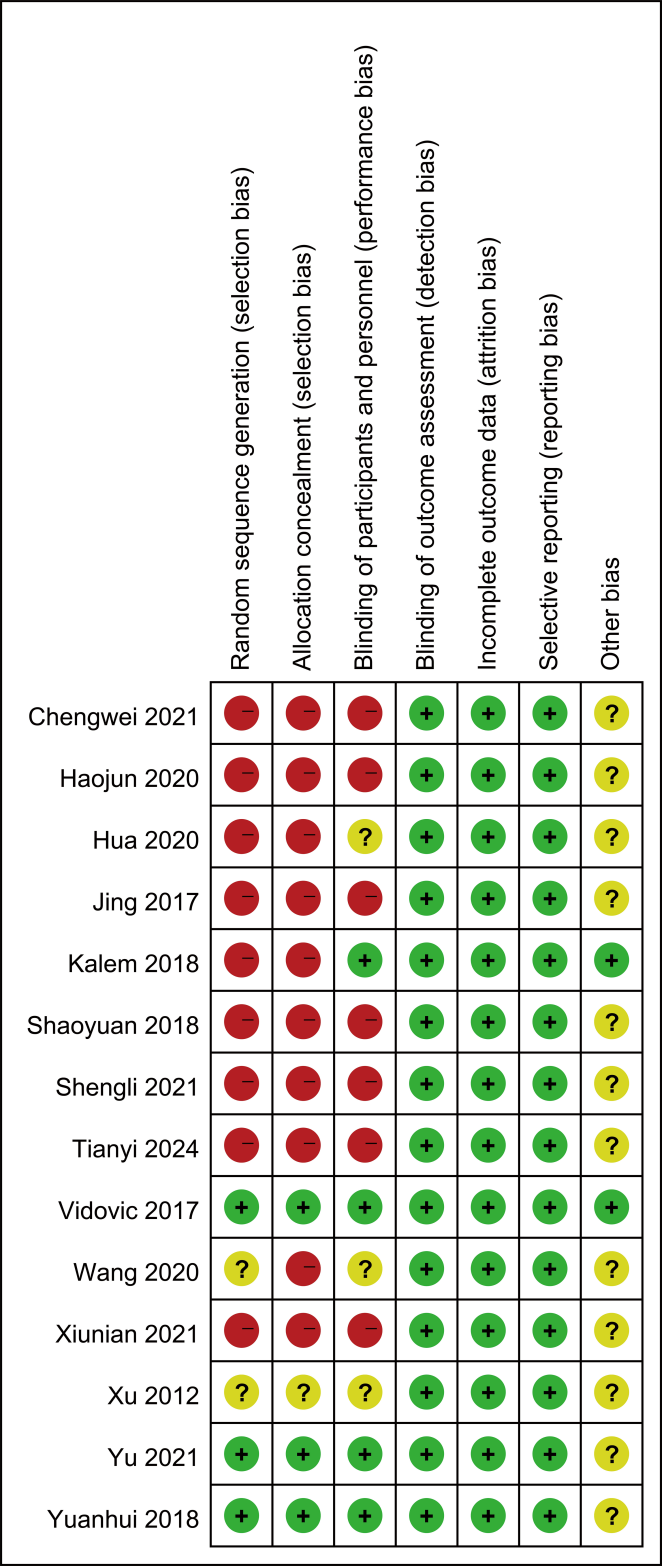
Risk of bias evaluation of included studies

The methodological quality of the included studies was evaluated according to the Cochrane Handbook “Risk of Bias Assessment Tool for Randomized Controlled Trials (version 5.1.0)”, as depicted in Figures 2, 3.

Meta-analysis

We conducted a comparison of the included literature based on four specific metrics to offer a more comprehensive and in-depth comparative evaluation of the therapeutic efficacy and safety of direct reduction internal fixation (PA) and indirect reduction internal fixation (AP) in the management of posterior ankle fractures. Among these studies, Wang [30] and Liu [34] stratified the sizes of posterior ankle fracture fragments into two distinct subgroups. Consequently, we extracted the data of these two subgroups separately. Moreover, the outcome metrics presented in Wang's 2020 [30] study were expressed as the median (P25, P75). However, for the purpose of achieving a more optimal and comprehensive analysis, we transformed these data into the format of mean ± standard deviation, referring to the methods described in the literature [36,37].

**Figure 4:**
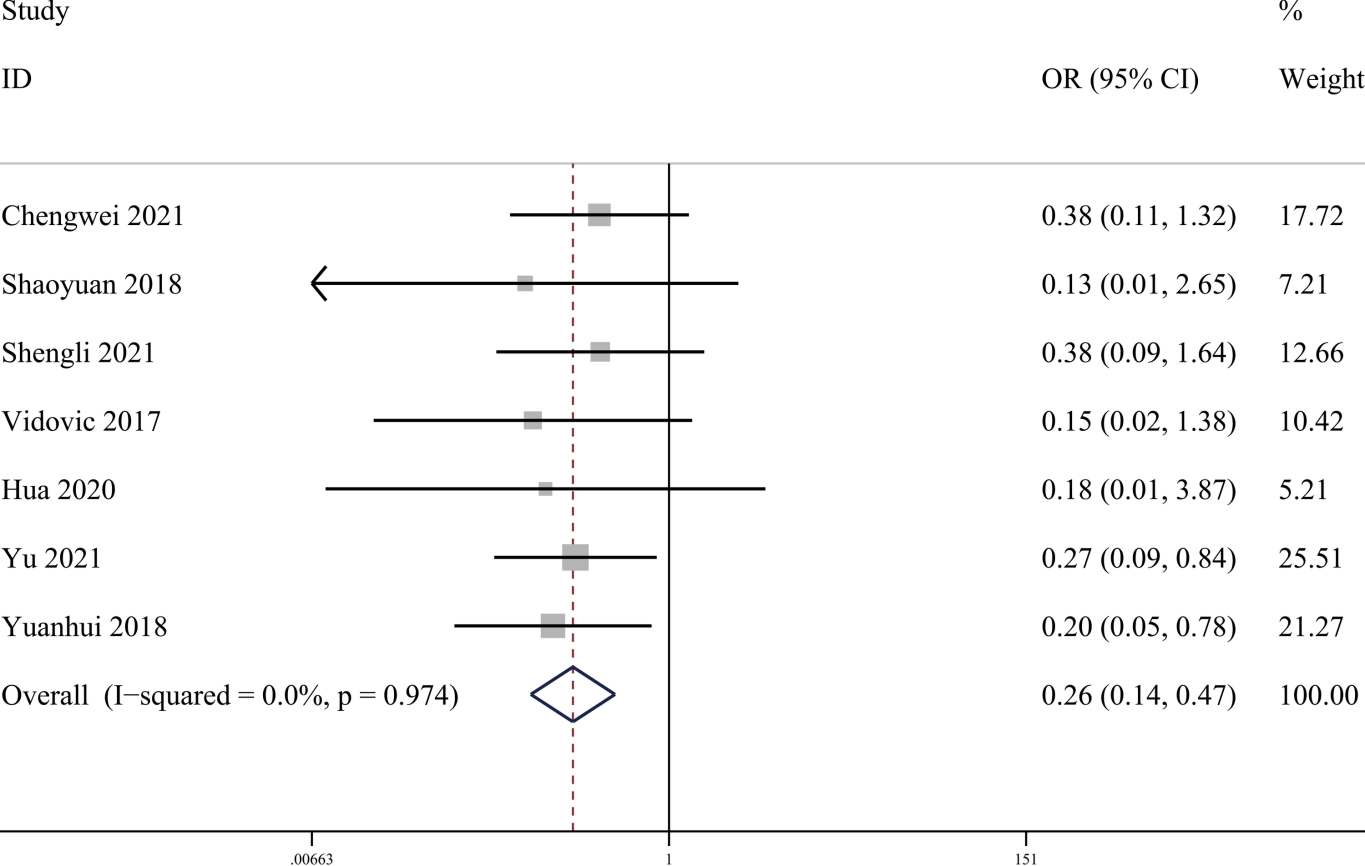
Forest plot comparing radiological evaluation >2mm of PA screws and AP screws PA: Posteroanterior; AP: anterior-posterior

**Figure 5:**
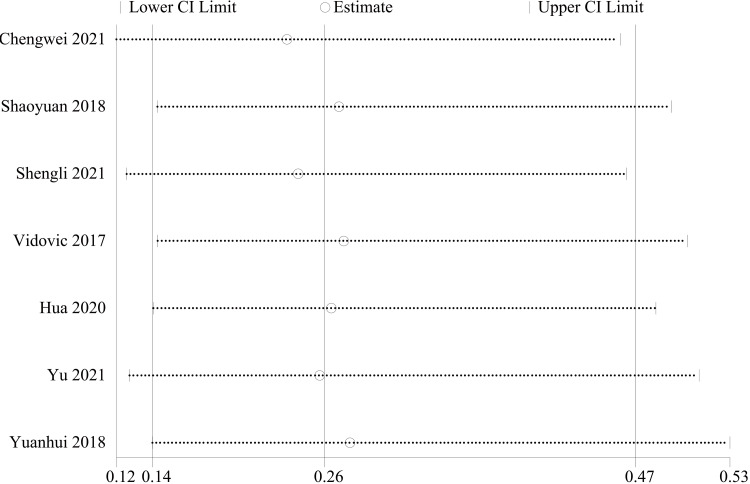
Sensitivity analysis chart for radiological evaluation >2mm

Imaging assessment: Seven publications [24-26,29,32,33,35] reported the index of postoperative imaging assessment of posterior malleolar fractures (radiologic assessment > 2 mm). There were 244 cases in the PA group and 241 cases in the AP group. The heterogeneity test showed I²=0.0%, p=0.974, and a fixed effect model was used. The results showed that the PA group had better fracture reduction imaging than the AP group (OR = 0.26, 95% CI [0.14, 0.47], p = 0.000; Figure 4). Sensitivity analyses performed on a study-by-study exclusion basis showed no significant change in the combined OR, indicating stable results and reliable conclusions (Figure 5).

**Figure 6:**
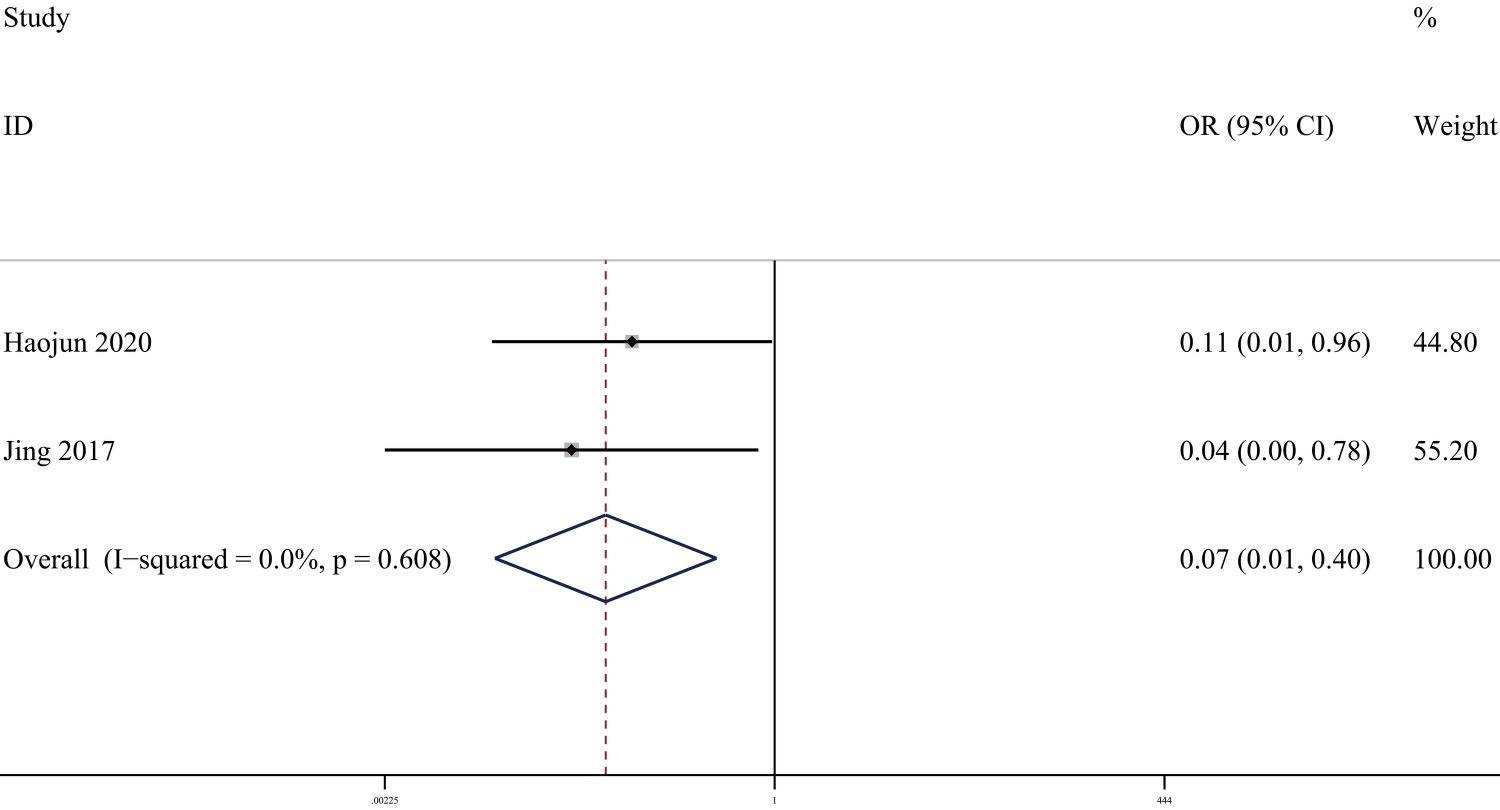
Forest plot comparing screw malpositioning of PA screws and AP screws PA: Posteroanterior; AP: anterior-posterior

**Figure 7:**
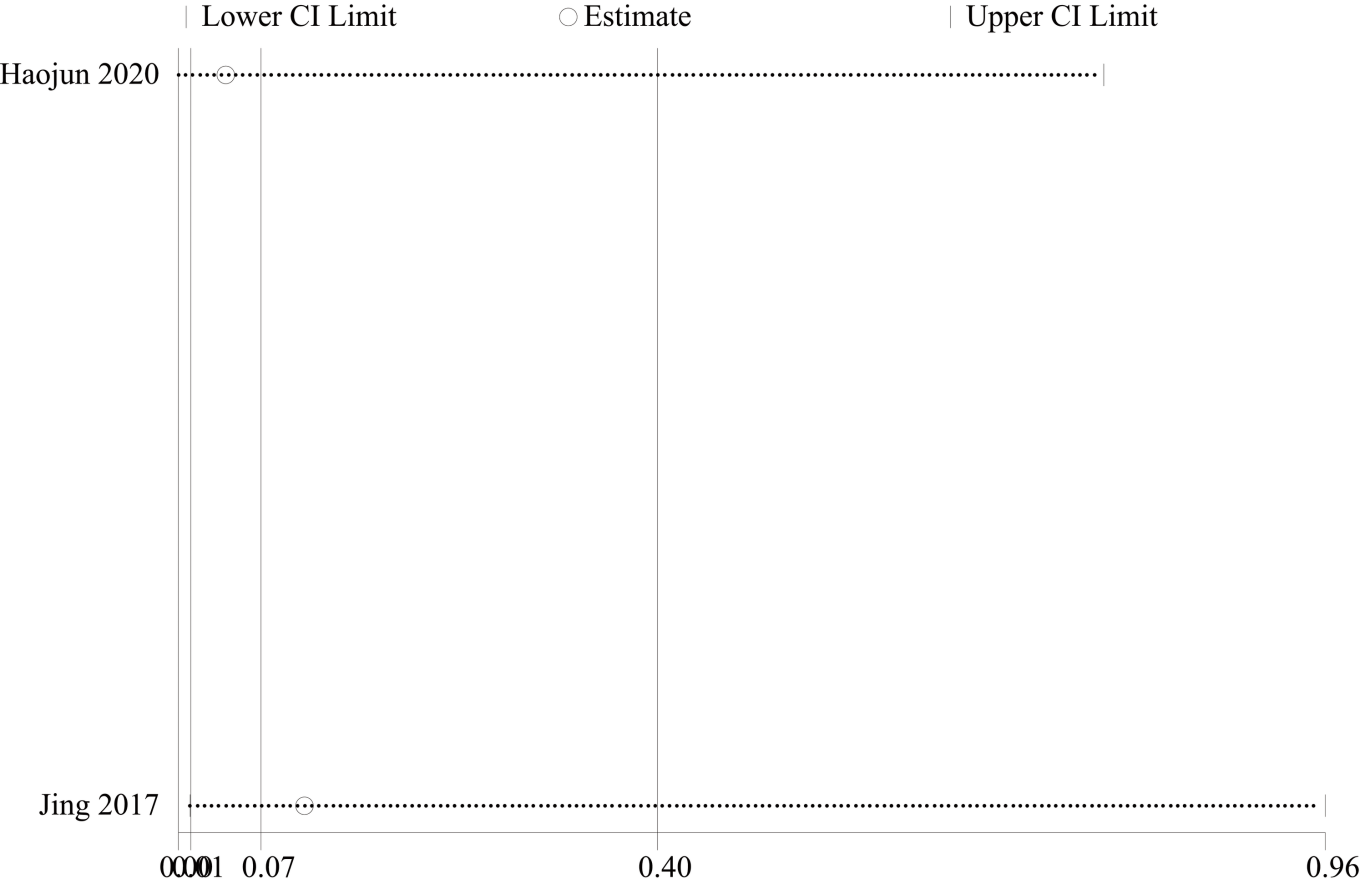
Sensitivity analysis chart for screw malpositioning

Screw malpositioning: Regarding the indicator of screw malpositioning after posterior malleolar fractures, we analyzed two papers [23, 28] that addressed this indicator. There were 61 cases in the PA group and 51 cases in the AP group. The heterogeneity test showed I²=0.0%, p=0.608, and a fixed effect model was used. The results showed that the precision of fracture reduction in the PA group was better than that in the AP group. (OR = 0.07, 95% CI [0.01, 0.40], p = 0.003; Figure 6). Sensitivity analyses performed on a study-by-study exclusion basis showed no significant change in the combined OR, indicating stable results and reliable conclusions (Figure 7). It is important to note that this conclusion is based on a very limited number of studies and events, which warrants cautious interpretation.

**Figure 8:**
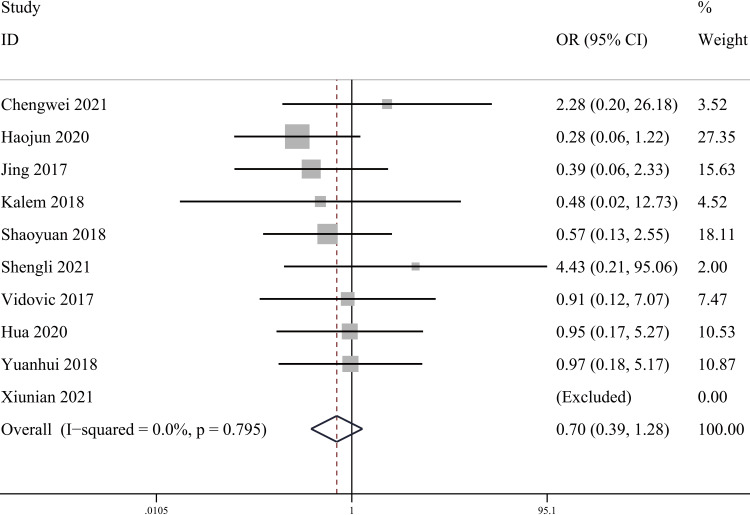
Forest plot comparing complications of PA screws and AP screws PA: Posteroanterior; AP: anterior-posterior

**Figure 9:**
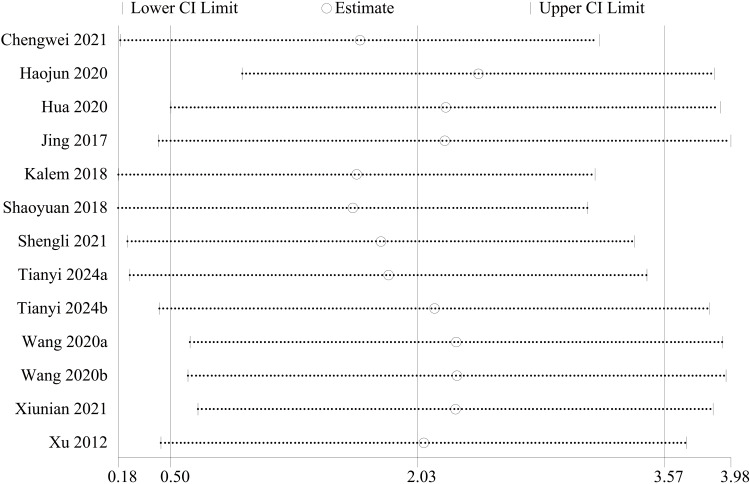
Sensitivity analysis chart for complications

**Figure 10:**
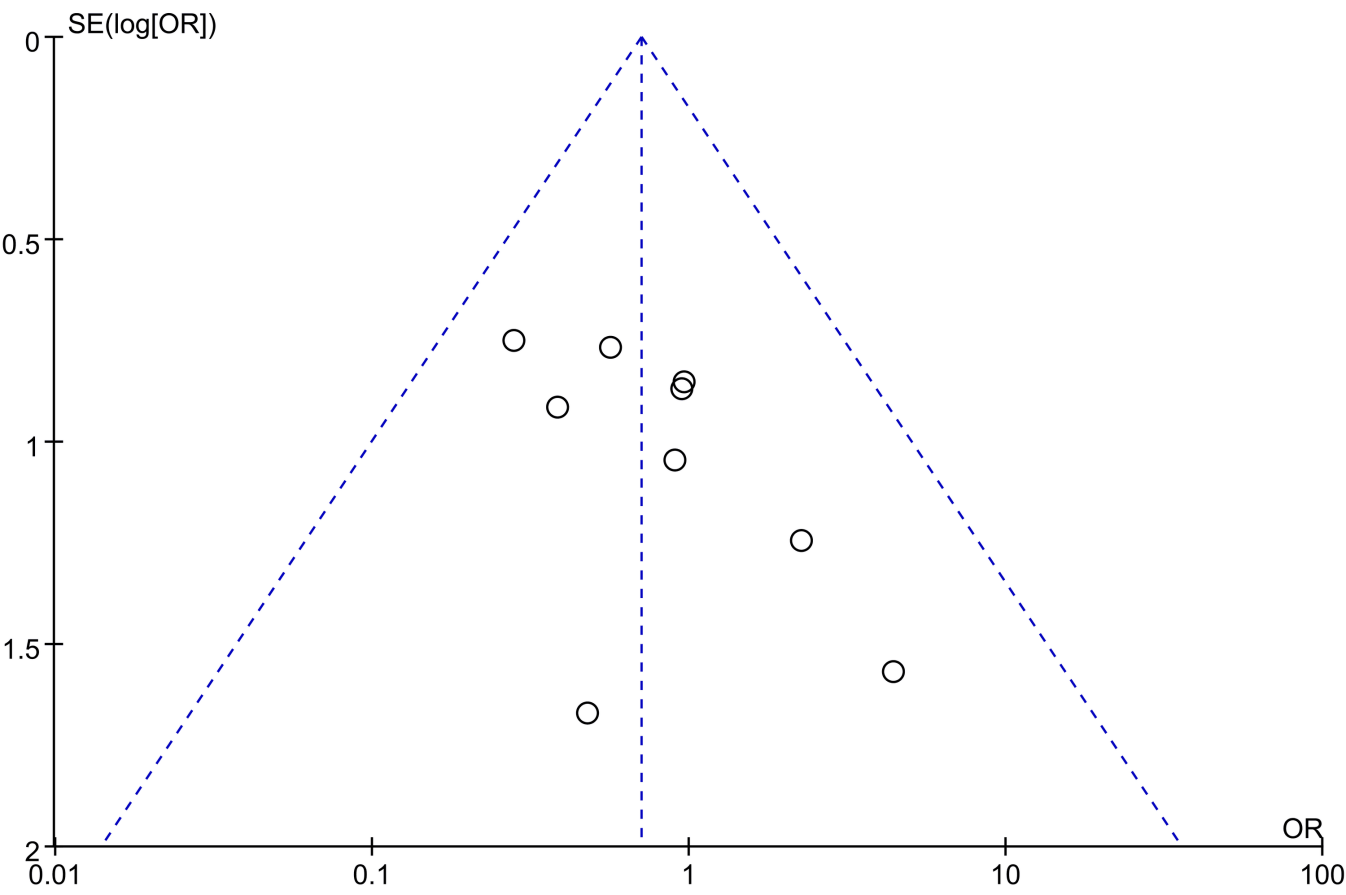
Funnel plot comparing Complications of PA screws and AP screws PA: Posteroanterior; AP: anterior-posterior

**Figure 11:**
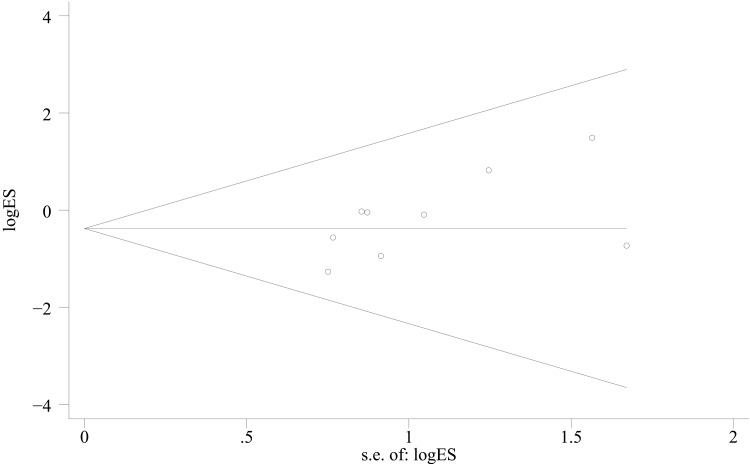
Begg’s funnel plot comparing complications of PA screws and AP screws PA: Posteroanterior; AP: anterior-posterior

**Figure 12:**
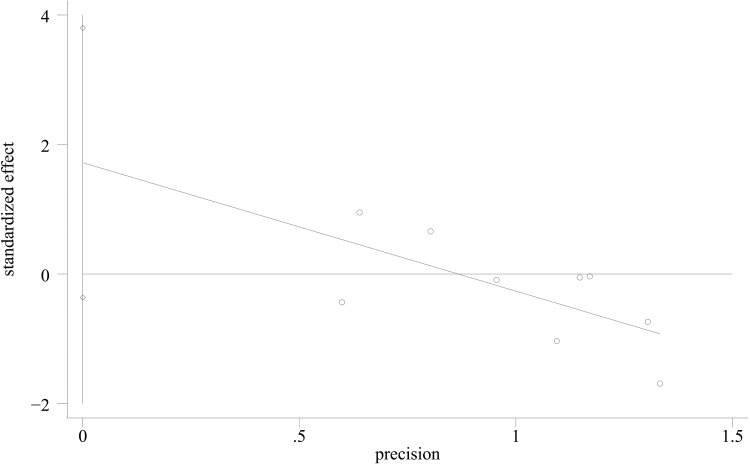
Egger’s publication bias plot comparing complications of PA screws and AP screws PA: Posteroanterior; AP: anterior-posterior

Complications: Regarding postoperative complications, 10 papers [23-29, 31, 33, 35] were used to compare the postoperative complications between the groups. There were 308 cases in the PA group and 294 cases in the AP group. The heterogeneity test showed I²=0.0%, p=0.795, and a fixed effect model was used. The results showed no significant difference in postoperative complications between the PA and AP groups. (OR = 0.70, 95% CI [0.39, 1.28], p = 0.252; Figure 8). The width of the confidence interval should be noted, as it cannot rule out a clinically important difference in either direction (potential Type II error). Sensitivity analyses performed on a study-by-study exclusion basis showed no significant change in the combined OR, indicating stable results and reliable conclusions (Figure 9). The funnel plot demonstrated that the distribution of points within the plot was nearly symmetric (Figure 10). Both Begg's test and Egger's test indicated the absence of publication bias, with P values of 0.175 for Begg's test and 0.092 for Egger's test (Figure 11 and Figure 12).

**Figure 13:**
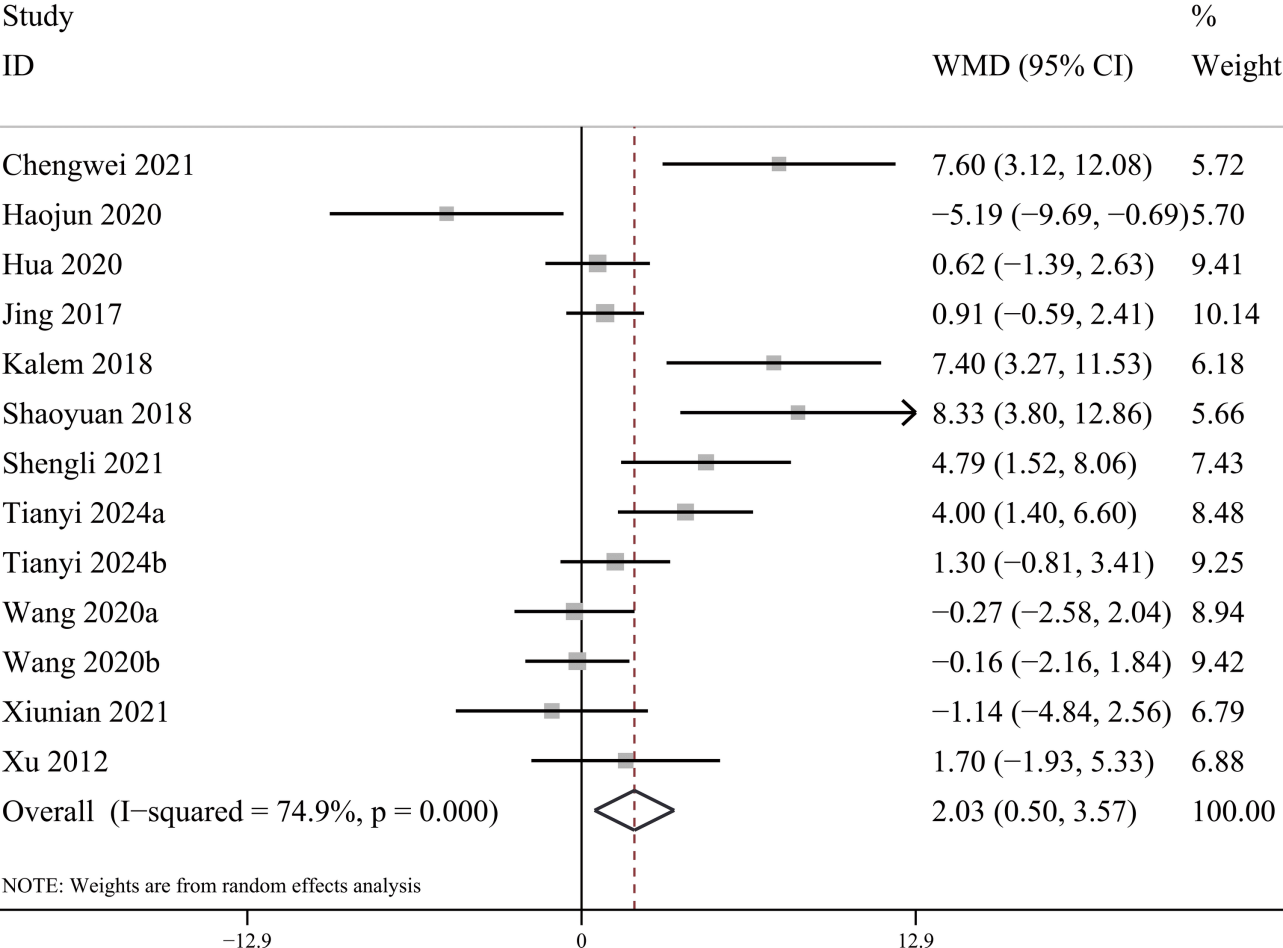
Forest plot comparing AOFAS scores for PA screws and AP screws PA: Posteroanterior; AP: anterior-posterior; AOFAS: American Orthopaedic Foot and Ankle Society

**Figure 14:**
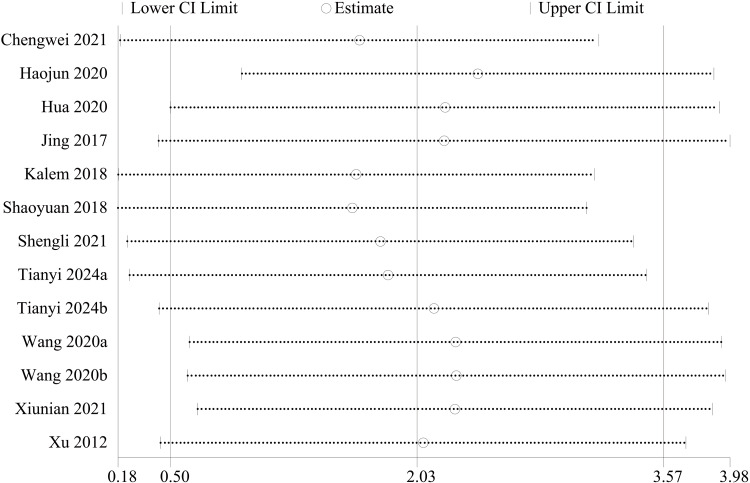
Sensitivity analysis chart for AOFAS scores AOFAS: American Orthopaedic Foot and Ankle Society

**Figure 15:**
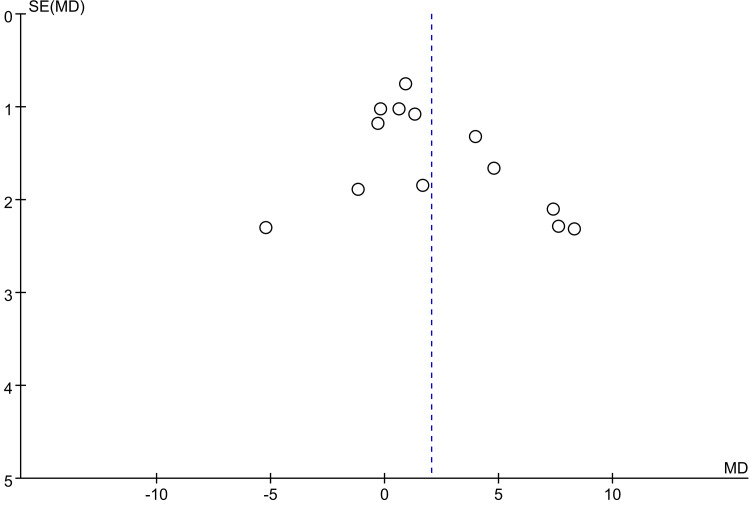
Funnel plot comparing AOFAS scores of PA screws and AP screws PA: Posteroanterior; AP: anterior-posterior; AOFAS: American Orthopaedic Foot and Ankle Society

**Figure 16:**
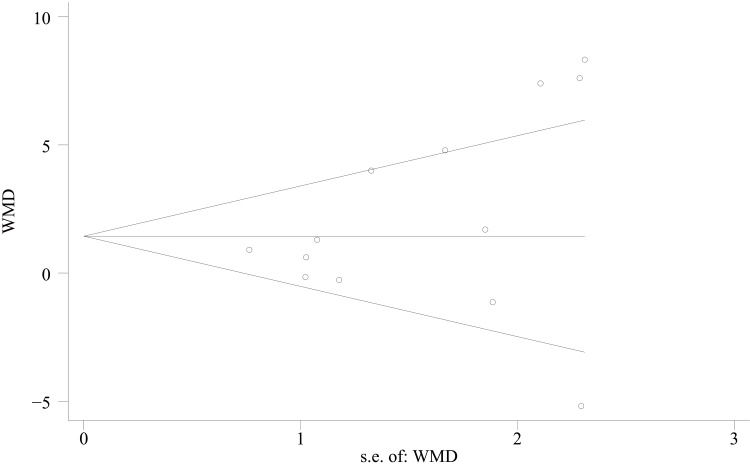
Begg’s funnel plot comparing AOFAS scores of PA screws and AP screws PA: Posteroanterior; AP: anterior-posterior; AOFAS: American Orthopaedic Foot and Ankle Society

**Figure 17:**
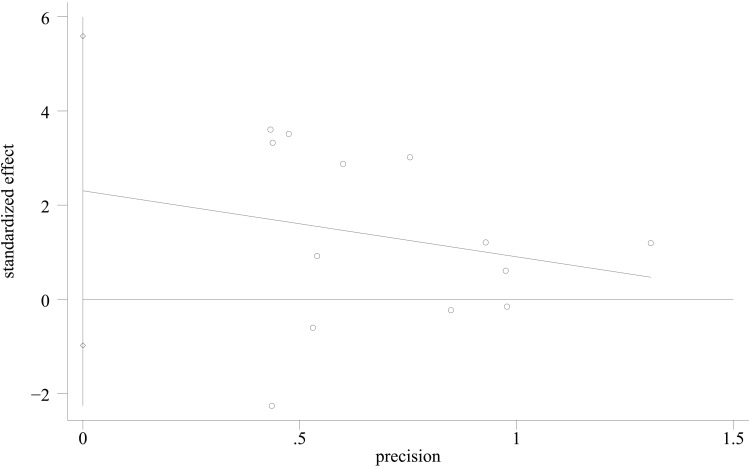
Egger’s funnel plot comparing AOFAS scores of PA screws and AP screws PA: Posteroanterior; AP: anterior-posterior

AOFAS score: For the functional assessment of the ankle joint after surgery, 11 papers [22-28,30,31,34,35] were included to compare the AOFAS scores. Of these, 575 cases were in the PA group and 492 cases in the AP group. The heterogeneity test showed I²=74.9%, p=0.000, so a random effects model was used. A statistically significant difference was found between the two groups (WMD = 2.03; 95% CI: 0.50, 3.57; P = 0.009, Figure 13). The considerable heterogeneity suggests underlying differences between studies that should be considered when interpreting this result. Furthermore, the observed difference of 2.03 points, while statistically significant, is below the range of commonly reported minimal clinically important difference (MCID) values for the AOFAS score (often cited as 8-12 points), which may limit its clinical relevance. Sensitivity analysis showed no significant change in the WMD composite value, indicating stable results and reliable conclusions (Figure 14). The funnel plot demonstrated that the distribution of points within the plot was nearly symmetric (Figure 15). Both Begg's test and Egger's test indicated the absence of publication bias, with P values of 0.100 for Begg's test and 0.151 for Egger's test (Figure 16 and Figure 17).

Discussion

This meta-analysis included 14 studies (three RCTs and 11 retrospective studies) involving 1,258 patients with posterior ankle fractures, systematically comparing the efficacy of PA screw fixation and AP screw fixation. The results demonstrated that the PA group exhibited significant superiority over the AP group in radiographic reduction quality (radiological parameters for displacement > 2 mm), incidence of screw malposition, and postoperative ankle functional recovery (AOFAS score), while no statistically significant difference was observed in postoperative complication rates between the two groups. However, the interpretation of these findings requires consideration of several important factors.

Firstly, the overall body of evidence is comprised predominantly of retrospective studies, which are prone to selection and confounding biases. The lack of high-quality RCTs is a significant limitation of the current literature on this topic.

Clinical Significance of Radiographic Reduction and Screw Placement

Anatomic reduction is the core objective in the treatment of ankle fractures, and the quality of reduction for posterior malleolar fragments directly influences the tibio-talar contact area and weight-bearing function [38,39]. In this study, the PA group showed a significant advantage in radiographic assessment of fractures with displacement > 2 mm (odds ratio [OR] = 0.26, 95% confidence interval [CI]: 0.14-0.47, P = 0.000), indicating that direct reduction via PA, performed under direct visualization, enables more precise anatomic reduction of fracture fragments and screw fixation, reducing risks of under-reduction or screw malposition associated with indirect reduction. Screw malposition may lead to internal fixation failure, soft tissue irritation, or nerve injury [12,14,23,28]. The lower incidence of screw malposition in the PA group (OR = 0.07, 95% CI: 0.01-0.40, P = 0.003) further confirms its technical advantages, consistent with previous findings that "direct reduction improves screw placement accuracy" [12,14].

Correlation Between Functional Recovery and Anatomic Reduction

The PA group demonstrated significantly better postoperative AOFAS scores than the AP group, reflecting superior functional recovery of the ankle joint. However, the mean difference of 2.03 points, while statistically significant, is of uncertain clinical importance as it falls below commonly accepted MCID thresholds. This suggests that the radiographic advantages of PA fixation may not translate into a substantial patient-perceived functional benefit in the short-to-medium term. This outcome is closely linked to the critical role of anatomic reduction-reconstruction of ankle biomechanical stability serves as the foundation for functional rehabilitation [40-46]. The PA technique, through direct reduction and precise fixation, effectively minimizes chronic pain and range-of-motion limitations caused by articular surface irregularities. Notably, the AP group’s reliance on intraoperative fluoroscopy and closed manipulation for indirect reduction may limit its ability to address subtle articular surface displacements, potentially increasing the long-term risk of post-traumatic arthritis [47-52].

Balance of Complication Risks

Although the PA group requires a posterolateral approach for fracture exposure, theoretically associated with risks of peroneal nerve injury or tendon impingement [53], this study found no significant intergroup differences in complication rates (e.g., wound infection, internal fixation failure). It is important to note that the confidence interval for this comparison was wide (OR = 0.70, 95% CI [0.39, 1.28]), indicating that our analysis may have been underpowered to detect a clinically relevant difference in complication rates, should one exist. This may be attributed to the proficiency of surgeons in the included studies with the PA technique, suggesting that standardized surgical protocols can effectively mitigate potential risks of direct approaches.

Study Limitations and Future Directions

Our study has several limitations. First, the search strategy, while specific, may not have captured all relevant literature due to its focused terms. Second, the restriction to English and Chinese languages may have introduced language bias. Third, the small number of studies reporting on certain outcomes (e.g., screw malpositioning) limits the strength of conclusions for those endpoints. Fourth, the substantial heterogeneity in AOFAS scores could not be fully explained, and the transformation of median values to means may have introduced imprecision.

The high proportion of retrospective studies (11/14) in this analysis may introduce selection bias and confounding factors. Additionally, heterogeneity existed across studies in fracture classification, follow-up duration, and functional assessment details. Although a random-effects model was used to address high heterogeneity, the stability of some results requires cautious interpretation. Future research should prioritize multicenter, large-sample RCTs to clarify efficacy differences between PA and AP techniques in specific fracture types (e.g., comminuted fractures, osteochondral injuries) and long-term outcomes such as the incidence of post-traumatic arthritis. Future studies should also strive for standardized reporting of outcomes, particularly radiographic parameters and complication definitions.

## Conclusions

This meta-analysis indicates that compared with the AP screw fixation technique, the PA screw fixation technique has significant advantages in aspects such as the quality of imaging reduction, the accuracy of screw implantation, and the functional recovery of the ankle joint when treating posterior ankle fractures, and it does not increase the risk of postoperative complications. However, the clinical importance of the small difference in functional scores is uncertain. Nevertheless, the percutaneous AP screw fixation technique is still widely used in clinical practice. Therefore, in clinical practice, surgeons can individualize their selection of the fixation method according to the type of fracture, the conditions of the soft tissues, and their own technical advantages, and give priority to direct reduction to achieve anatomical reconstruction, so as to improve the long-term prognosis of patients. Higher-quality studies are also needed to further verify the long-term efficacy of these two techniques and the differences in their indications.
